# Human Tracking in Top-View Fisheye Images: Analysis of Familiar Similarity Measures via HOG and against Various Color Spaces

**DOI:** 10.3390/jimaging8040115

**Published:** 2022-04-16

**Authors:** Hicham Talaoubrid, Marina Vert, Khizar Hayat, Baptiste Magnier

**Affiliations:** 1 EuroMov Digital Health in Motion, Univ Montpellier, IMT Mines Ales, 30100 Ales, France; marina.vert@mines-ales.org (M.V.); baptiste.magnier@mines-ales.fr (B.M.); 2 College of Arts and Sciences, University of Nizwa, Nizwa 616, Oman; khizar.hayat@unizwa.edu.om

**Keywords:** color spaces, similarity functions, fisheye

## Abstract

The purpose of this paper is to find the best way to track human subjects in fisheye images by considering the most common similarity measures in the function of various color spaces as well as the HOG. To this end, we have relied on videos taken by a fisheye camera wherein multiple human subjects were recorded walking simultaneously, in random directions. Using an existing deep-learning method for the detection of persons in fisheye images, bounding boxes are extracted each containing information related to a single person. Consequently, each bounding box can be described by color features, usually color histograms; with the HOG relying on object shapes and contours. These descriptors do not inform the same features and they need to be evaluated in the context of tracking in top-view fisheye images. With this in perspective, a distance is computed to compare similarities between the detected bounding boxes of two consecutive frames. To do so, we are proposing a rate function (S) in order to compare and evaluate together the six different color spaces and six distances, and with the HOG. This function links inter-distance (i.e., the distance between the images of the same person throughout the frames of the video) with intra-distance (i.e., the distance between images of different people throughout the frames). It enables ascertaining a given feature descriptor (color or HOG) mapped to a corresponding similarity function and hence deciding the most reliable one to compute the similarity or the difference between two segmented persons. All these comparisons lead to some interesting results, as explained in the later part of the article.

## 1. Introduction

**Figure 1:**
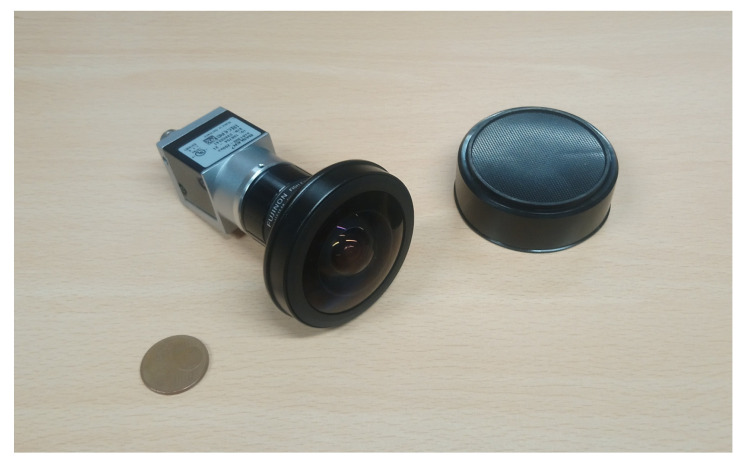
Basler ace acA2040-120uc color camera equipped with a fish eye lens utilized in our experiments. It is positioned next to a EUR 0.05 coin.

**Figure 2:**
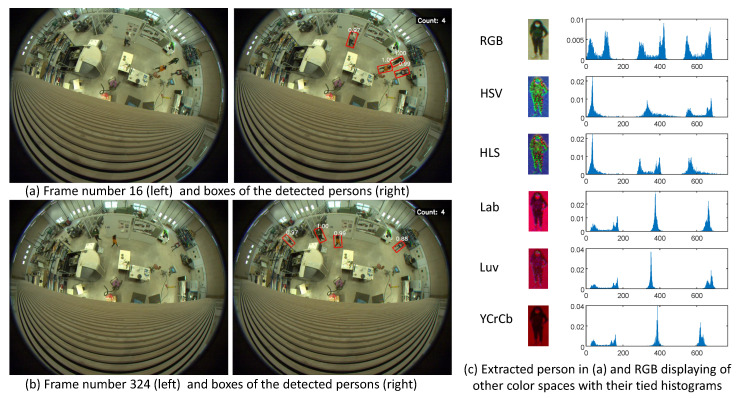
Persons extracted from fisheye images and displaying of different color space representation with their tied histograms.

In the computer vision domain, detecting and tracking people constitutes an important area [1]. Significant approaches exist to detect pedestrians in monocular images [2]. Recently, numerous papers have been published on people or pedestrian detection using deep learning techniques. In this paper, our aim is to estimate the similarity between several small images containing persons by extracting color features. Indeed, color spaces have a “low computational” cost and must be investigated to choose the ideal space for a specific application [3,4,5,6]. The proposed study focuses on the extraction of people’s bounding boxes from top-view fisheye images. Usually, with its specific lens (see Figure 1), a fisheye camera offers panoramic views of 2π radian angles [7,8]. Therefore, objectives with wide-angle lenses capture images that are typically warped, as illustrated in Figure 2. Besides the deformations, a challenging task is to tackle the fact that images may differ significantly due to variations in factors like illumination and human posture. Moreover, in the top view, with the camera axis pointing vertically, people standing straight may appear oriented in the image, pointing towards the image’s center due to the distortion of the camera lens. Therefore, a conventional perspective human detection technique such as the histogram of oriented gradients (HOG) cannot be directly used [9]. Various methods [10,11,12,13,14] have been implemented in the literature for distorted perspectives but they do not enable the tracking of the movement of a person throughout the video acquired by a fisheye camera, especially in a top view perspective.

In this paper, a comparison of the HOG and different usual color spaces with different similarity functions for histograms is presented in the context of human segmentation tracking via a fisheye camera. This communication is the continuation of our previous paper [15], in which only two color spaces are compared with only one similarity function. Our main objective is to determine the best combination of the similarity functions and color spaces that will enable better tracking of segmented persons via color features. The detection part is performed via a deep learning algorithm presented in [12]. Finally, experiments are carried out by OpenCV software and a function S enables an objective comparison of the results.

The rest of the paper is organized as follows. The next section presents the different color spaces. Then Section 3 is devoted to detailing the HOG. The similarity functions are introduced in Section 4. Finally, Section 5 presents the experimental protocol and different results before conclude this paper in Section 6.

## 2. Colorimetric Spaces

Standard color images are represented with red, green, and blue channels (RGB). Typically, a digital color image has a minimum of 24 bits/pixel, with 8 bits assigned to each color component of the three-color channels. Consequently, a color image can contain 65,536 different color shades. RGB is a standard color space to represent images on displays, cameras, projectors, etc. Although RGB is the primary color space used to describe the spectral content of color signals, a variety of other representations have been developed, as detailed in [16]. Most of them are summarized in [17]. Their use is focused on different image classification areas: face detection, food quality (fruits, vegetables, wine, honey...), medical images, and scene understanding are important to note, among others.

Some of these representations are more appropriate for image processing than others. The objective of this paper is to optimize color space/distance pair for better pedestrian tracking with fisheye cameras. We will limit this preliminary study to six well-known color spaces outlined in Table 1 (also see Appendix A) with special reference to the involvement of the HOG. The following subsections describe four types of these color spaces.

### 2.1. The Additive Models: RGB

As introduced above, the mixture of the three primary colors (red, green, and blue) allows the production of all the color shades perceptible to the human eye. Considering a color image representation where each channel is coded with 8 bits, the RGB space offers a model in which each pixel is represented by a triplet (R, G, B) with the intensity of each pixel component ranging from 0 to 255. Note that OpenCV reads images in BGR format (instead of RGB) but the treatments are the same as in the presented study.

### 2.2. The Natural Models: HSV and HLS

The RGB space can be visualized as a color cube, considering the basis is formed by the red, green, and blue vectors. Cylindrical color models that remap the RGB primary colors into dimensions are easy to understand visually. Indeed, HSV and HSL models are called natural because they are closer to the human perception of colors. In these models each pixel is coded by a triplet (H, S, V) or (H, L, S); for both spaces, the value H represents the hue. Hue is defined as the dominant wavelength of a source. Consider a “color wheel” around which we concatenate all the colors, so each primary color would be spaced 120${}^{\circ }$, the hue is represented by an angle (between 0 and 360${}^{\circ }$), tied to the color of the pixel. As images are coded in 8 bits, so we will take for H half of its real value (so that its value is between 0 and 255). The S value, also common to both spaces, represents the saturation; it expresses the color contrast. It is between 0 and 255 (0 corresponds to gray whatever the color hue, and 255 a point on the color wheel). The last element of the triplet corresponds to the lightness and value (V or S): the higher it is, the clearer the image is.

### 2.3. The Human Vision Models: L*a*b* and L*u*v*

Many mixtures in the RGB space give indistinguishable colors. The International Commission on Illumination (CIE) has introduced three-dimensional representations, (including L*a*b* and L*u*v*, which we will study) that take the human perception for reference. Indeed, for a point of these spaces, L corresponds to the lightness, whereas a and b (or u and v) represent the chroma coordinates. L*a*b* and L*u*v* spaces are quite similar, a and b (or u and v) coordinates measure positions along with the green/red and blue/yellow axes. These spaces are difficult to understand intuitively. Since we use images coded in 8 bits, the values of each element of the triplet coding each pixel are reduced between 0 and 255.

### 2.4. The Television Models: YCbCr/YUV

The YCbCr model is one of the models used for digital or analog compression. The Y value represents the luminance that describes the image in black and white and the values (Cr, Cb) code the chrominance. Cr describes the red–green difference and Cb the blue–yellow difference. The Y component is more sensitive to the human eye, it must remain precise to distinguish colors correctly. However, Cb and Cr are less sensitive to the human eye, so they do not need to be more accurate. That is why this space is useful in image compression; especially in the JPEG algorithm.

Table 1 summarizes the different acronyms of the utilized color spaces, along with their ranges for OpenCV implementation. The appendix at the bottom recalls the way to compute each color space from RGB. Further, Figure 2 shows a sub-image of a segmented person under different color spaces.

Having described all the types of color spaces, we will now introduce the other feature descriptor that we will be comparing, namely the HOG.

## 3. Histogram of Oriented Gradients (HOG)

Contemporary machine learning hinges largely on what is now widely known as feature engineering, which simply means to derive newer features, from those in hand, for the refinement of the underlying model. With a considerable amount of data involved in multimedia, especially images and videos, the available raw features need to be ‘compressed’ to a simplified representation called the feature descriptors. In object detection, an important descriptor is the HOG, which relies on the shape and the contours of the object. Unlike simple edge descriptors, HOG is dependent on the orientation, in addition to the magnitude of the gradient. In practice, the image or the frame could be partitioned into regions, and HOG is computed for each region separately. As explained in [9], a typical edge detection via gradient for a grey level image involves the following steps:

1. Smooth the input image (f(x,y)), e.g., via a Gaussian function (G(x,y)) to get $\hat{f}\left(x,y\right)$:
  (1) $$\hat{f}\left(x,y\right)=f\left(x,y\right)*G\left(x,y\right).$$
2. Compute the *x* and *y* derivatives of $\hat{f}\left(x,y\right)$
  (2) $${\hat{f}}_{x}\left(x,y\right)=f\left(x,y\right)*D_{x}\left(x,y\right).$$
  (3) $${\hat{f}}_{y}\left(x,y\right)=f\left(x,y\right)*D_{y}\left(x,y\right).$$
  where $D_{x}$ and $D_{x}$ are the kernels for first order derivative in *x* and *y* directions, respectively. The familiar ones are Robert, Sobel, and Prewitt, $\left[-1\;0\;1\right]$ masks, etc. Such masks enables a computation of precise locations of edges of small objects and their tied orientations (see evaluation of edge detectors in [18]).
3. The magnitude of gradient magn(x,y) is given by:
  (4) $$magn\left(x,y\right)=\sqrt{{\hat{f}}_{x}^{2}\left(x,y\right)+{\hat{f}}_{y}^{2}\left(x,y\right)}.$$
  A simplified version is however preferred, which is:
  (5) $$magn\left(x,y\right)=|{\hat{f}}_{x}\left(x,y\right)|+|{\hat{f}}_{y}\left(x,y\right)|.$$
4. For each pixel, the direction of gradient θ is computed as:
  (6) $$\theta \left(x,y\right)=\tan^{-1}\frac{{\hat{f}}_{y}\left(x,y\right)}{{\hat{f}}_{x}\left(x,y\right)}.$$

The resultant magnitude (magn(x,y)) and orientation (θ) maps can be used to compute HOG descriptors.

A simple approach to rely only on the orientation map and realize the histogram is to make a frequency table based on the orientation angles, either individually or in bins. However, the magnitude map can also be brought into play, if one replaces the frequency with the magnitude, against each angle (or a bin thereof). In such a case, a bin approach might be more feasible, but a given magnitude may have two adjacent candidate bins; it is better to assign to the nearest neighbor or divide among the bins based on nearness.

Usually, each image map will be partitioned to small fix-sized dyadic cells (8×8 or 16×16 or more) and HOG descriptors would be computed for each cell from its orientation map (in correspondence to its magnitude map, if needed). In essence, each of the cells has its own histogram and HOG descriptor. Better normalize the HOG descriptors, for better results, by dividing each bin value by the square root of the sum of the squares of all the bin values for the cell.

The HOG descriptor is usually a vector (k×1 matrix) of the form $\left[b_{1},b_{2},\ldots .,b_{k}\right]$, where *k* is the number of bins and $b_{i}$ denotes the value in *i*th bin. A normalized vector for a given cell could be:

(7) $$\left[\frac{b_{1}}{p},\frac{b_{2}}{p},\ldots .,\frac{b_{k}}{p}\right],$$

where

(8) $$p=\sqrt{b_{1}^{2}+b_{2}^{2}+\ldots .b_{k}^{2}}.$$

An m×n image, if divided into w×w blocks would have a total of m/w×n/w = $mn/w^{2}$ blocks. Since each block has a vector of k×1 features, we end up with $k\times mn/w^{2}$ features in total.

The HOG is useful to recognize people and objects in images [9]; it could be also combined with different strategies for the tracking process [19]. Consequently, the aim of this paper is to investigate if the HOG enables more precise histogram-based tracking of persons using a top-view moving fisheye camera than color spaces. In this study, we chose to implement the HOG on full gray-scale images, we used the *BGR2GRAY* OpenCV function to convert color images into scalar images. Considering this function, for each pixel tied to the (B, G, R) component of a color image, the pixel Y corresponding to the gray-scale image is computed such that:

(9) Y=0.299·R+0.587·G+0.114·B.

**Figure 3:**
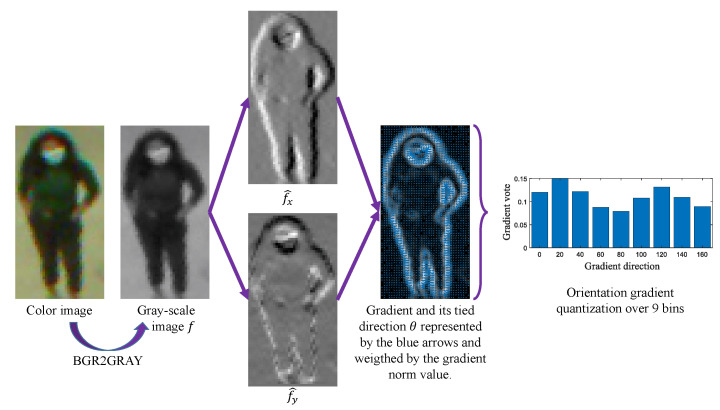
HOG computation on a full gray-scale image using [−101] and ${\left[-1\;0\;1\right]}^{t}$ masks.

greenNote that the filters we used to implement the HOG are [10−1] and ${\left[1\;0\;-1\right]}^{t}$ and the gradient magnitude is computed with Equation (4); greensee the whole description in Figure 3.

After the description of the feature descriptors studied in this paper, now let us look at similarity functions.

## 4. Similarity Functions: Our Approach to Compare Histograms

This work is based on the comparison of HOG and six different color spaces, as a function of each of the chosen six different distance measures. To this end, the input is a video from a fisheye camera that is captured under the assumption that a limited number (2–4) of people are moving under it. In brief, the following steps were involved:

(i) Employ the RAPiD method [12] to detect the different people in the frames of the video in the form of the bounding boxes.
(ii) Convert each bounding box from RGB space to each of the desired spaces.
(iii) In a given color space, compute the histograms $H_{1}$ and $H_{2}$ corresponding to the two histograms tied to two different images. They both contain *N* bins, and for an integer *k* between 0 and N−1, $H_{i}\left(k\right)$ represents the value of the *k*-th bin (for i∈{1,2})
(iv) Normalize the histograms (i.e., $\sum_{k=0}^{N-1}H_{i}\left(k\right)=1$) in order to enable an easy comparison of two images, even of different sizes.
(v) Compute the average of the histogram (${\bar{H}}_{i}$) which is easily computed (optional, see Table 2):
  (10) $${\bar{H}}_{i}=\frac{1}{N}\cdot \sum_{k=0}^{N-1}H_{i}\left(k\right).$$
(vi) With $H_{1}$, $H_{2}$, ${\bar{H}}_{1}$, and ${\bar{H}}_{2}$ as inputs, compute the identified six similarity measures (detailed bellow and in Table 2) for each of the chosen six color spaces.
(vii) Compute the identified six similarity measures for HOG.
(viii) Carry out a grid based comparison between the distance measures and color spaces as well as HOG using S function to decide the optimum.

Our comparison is based on six different similarity measures summarized in Table 2. These measures are in fact mostly distances and it will not be out of place to explain these briefly.

1. **Histogram Correlation:** The correlation measure, called correlation coefficient, is often used in statistics to determine if there is any interdependence between two random variables. It is defined as the quotient between their covariance and the product of their standard deviation. Originally, the result is between −1 and 1, where 1 indicates a perfect match and −1 a complete mismatch. To standardize with other distances, in particular, the complement of this measure is computed so that it translates to a value close to 0, for better matching.
2. **Chi-square ($\chi^{2}$) measure:** The chi-square ($\chi^{2}$) test statistic is a commonly used statistical measure to calculate the similarity between frequency distributions. The original chi-square measure, based on the Pearsons’s chi-square test statistic, is not only asymmetrical but also raises a singularity problem if either or both of the two histograms in the comparison contain a zero bit. That is why it was modified to nullify both the problems. This alternative version gives a measure of 0 for a perfect match and on the contrary, gives a score close to 1 for two very distant histograms (or images).
3. **Histogram Intersection:** The straightforward histogram intersection measure gives the proportion of pixels that have the same intensity. Therefore, a perfect match is obviously given by a score of 1. Like the correlation, the complement of this measure is being computed in this work.
4. **Bhattacharyya distance:** This measure estimates the similarity between two probability distributions. It has a geometric interpretation: considering two *n*-dimensional unit vectors p(i) and q(i), this distance can be interpreted as a cosine of the angle between vectors formed by taking the square root of each coordinate of the initial vectors i.e., the angle formed by $\sqrt{p(i)}$ and $\sqrt{q(i)}$. This measure is regularly used for object tracking [20].
5. **Kullback–Leibler divergence:** The Kullback–Leibler (KL) divergence is a measure of dissimilarity between two probability distributions. Considering two images, KL can be interpreted as a divergence by the amount of information lost when we approximate one image with another. So, a value close to 0 implies a good match and two very different images will have a high Kullback–Leibler divergence, often even higher than 1.
6. **Manhattan distance** The Minkovski distance is a generalization of the Manhattan distance. Let *X* and *Y* be two distributions, the general formula of the Minkovski distance can be expressed as D(X,Y)=$\sqrt[{n}]{\sum_{i}{\left[X(i)-Y(i)\right]}^{n}}$. In this paper, the choice is to limit to the first order, also called the $\ell_{1}$ norm, corresponding to the Manhattan distance.

We have now explained all the notions necessary for our study. We can now move on to the experiments section.

## 5. Experimental Comparisons

Before tackling the experimental protocol, here is an example of histogram-based comparisons.

### 5.1. Example of Histogram-Based Comparisons

Table 3 shows an example of a comparison between several images containing different persons. In most cases, the lowest distance is the distance between the person $P_{1}$ at the time *t* and itself at the time t+x, but sometimes it is not the case. As an example, when the Bhattacharyya distance is used with the CIE L*a*b* color space, the person $P_{3}$ at the time *t* is closer to the person $P_{1}$ at the time t+x than the person $P_{1}$ itself at the time *t*. This shows that some associations are better than others and that it is important to choose the color space/distance couple well for better results. That is the purpose of this paper.

### 5.2. Experimental Protocol

To compare the HOG with different color spaces and the different distance measures, respectively presented in Table 1 and Table 2, a number of videos were used. These videos were all taken with a Basler ace acA2040-120uc (https://www.edmundoptics.com/p/basler-ace-aca2040-120um-monochrome-usb-30-camera/34668/, accessed on 28 March 2022) color camera equipped with a fisheye lens (https://www.edmundoptics.co.uk/p/23quot-format-c-mount-fisheye-lens-18mm-fl/16922/, accessed on 28 March 2022), see Figure 1 for a picture of the device; some features are given in Table 4. The scenario pertains to around 2 to 4 people walking and moving under the camera. The Rotation-Aware People Detection in Overhead fisheye Images (RAPiD) method [12] is used to detect different people in the frames. Indeed, the RAPiD method predicts bounding boxes of people, with a certain center, size, and angle of the bounding box. Even though it is useful for several other tasks, only color features inside the bounding boxes in this study interest us. All the bounding boxes initially in the RGB color space were converted into all the color spaces that are mentioned above. We then computed the different distances between the bounding boxes of the consecutive frames.

**Figure 4:**
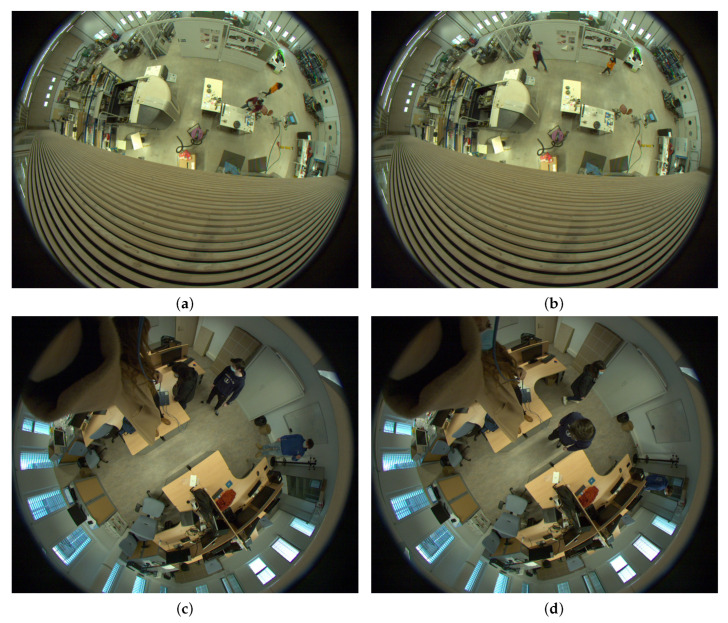
Extracted frames from videos 2 and 3 with two and three persons respectively. (**a**) Video 2, frame 194. (**b**) Video 2, frame 494. (**c**) Video 3, frame 94. (**d**) Video 3, frame 192.

For the sake of discussion, we are relying on three videos from our sample; two of them were taken in a hall, as shown in Figure 2 and Figure 4a,b. In the first two videos, there are respectively four and two people walking simultaneously that we want to track. We can see that the RAPiD method is quite robust and detects people, even when the view is quite occluded and people appear unusually smaller in the scene. The third video was taken in a classroom and shows three people walking simultaneously. The camera is much closer this time, and once again, the RAPiD method can detect people, even when they are walking right along the optical axis of the camera, as shown in Figure 4c,d.

However, sometimes, because of a cluttering background or dark lighting, not everyone is detected. As a result, to ensure that all the people were correctly detected, in order to use all the bounding boxes, only the frames where the RAPiD detected the right number of people are kept (*k* bounding boxes for a video with *k* segmented persons, with *k* ∈ {2,3,4}).

### 5.3. Comparison with Multiple Video Acquisitions

**Figure 5:**
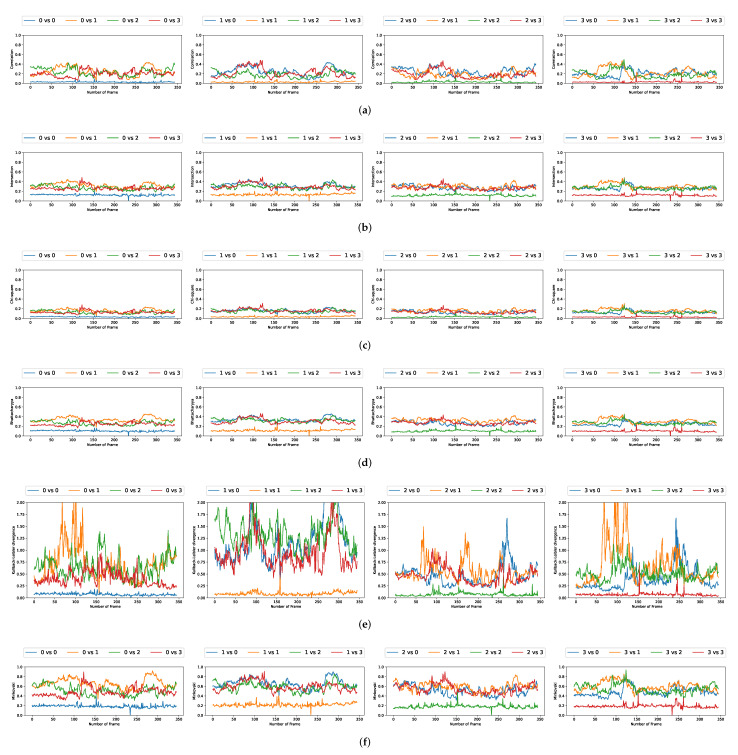
Curves comparing persons from one frame to another in video 1 (persons 0, 1, 2, and 3). (**a**) RGB and Correlation. (**b**) HLS and Intersection. (**c**) HSV and Chi-square. (**d**) L*a*b and Bhattacharyya. (**e**) L*u*v and KL divergence. (**f**) YCrCb and Minkovski.

**Figure 6:**
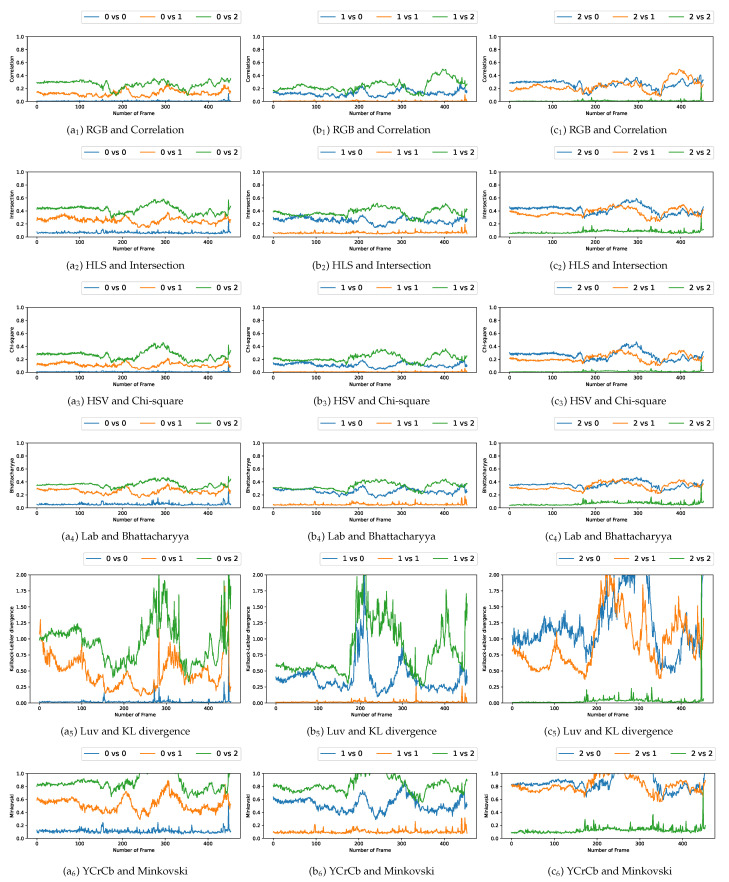
Curves comparing persons from one frame to another in video 2 (persons 0, 1 and 2).

**Figure 7:**
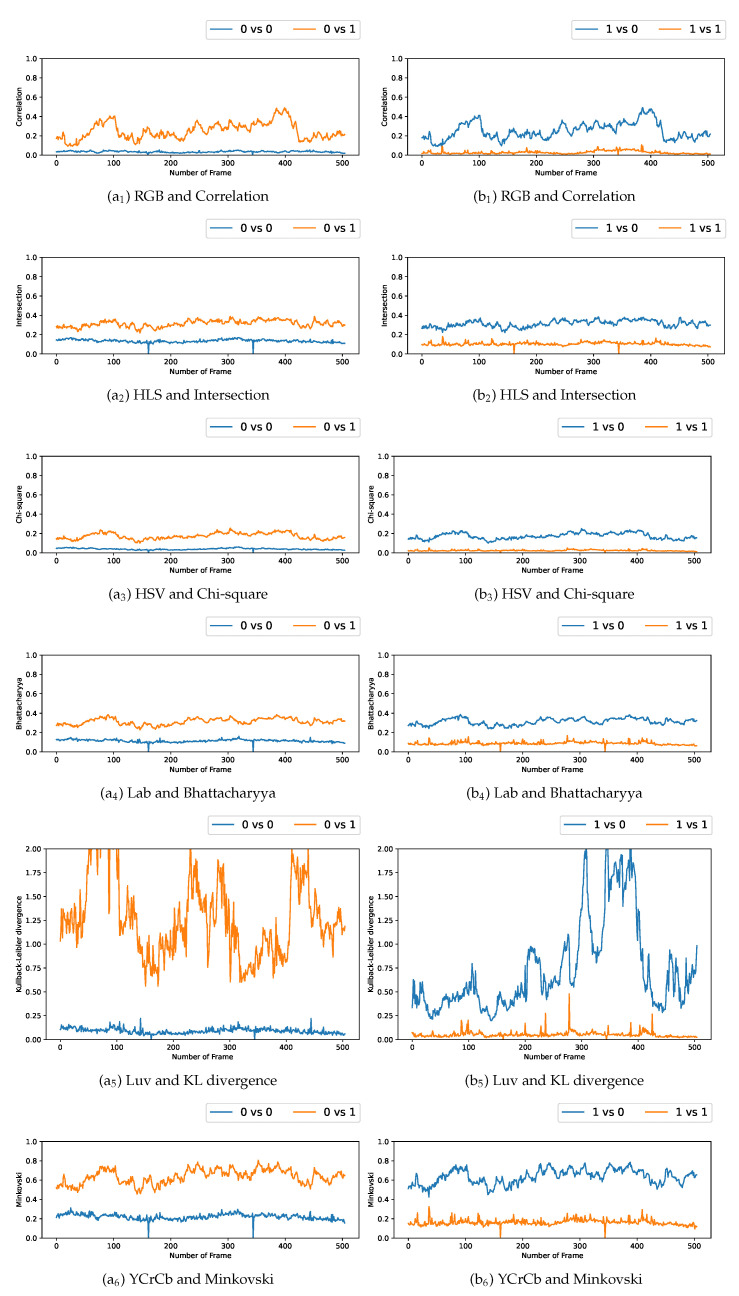
Curves comparing persons from one frame to another in video 3 (persons 0 and 1).

**Figure 8:**
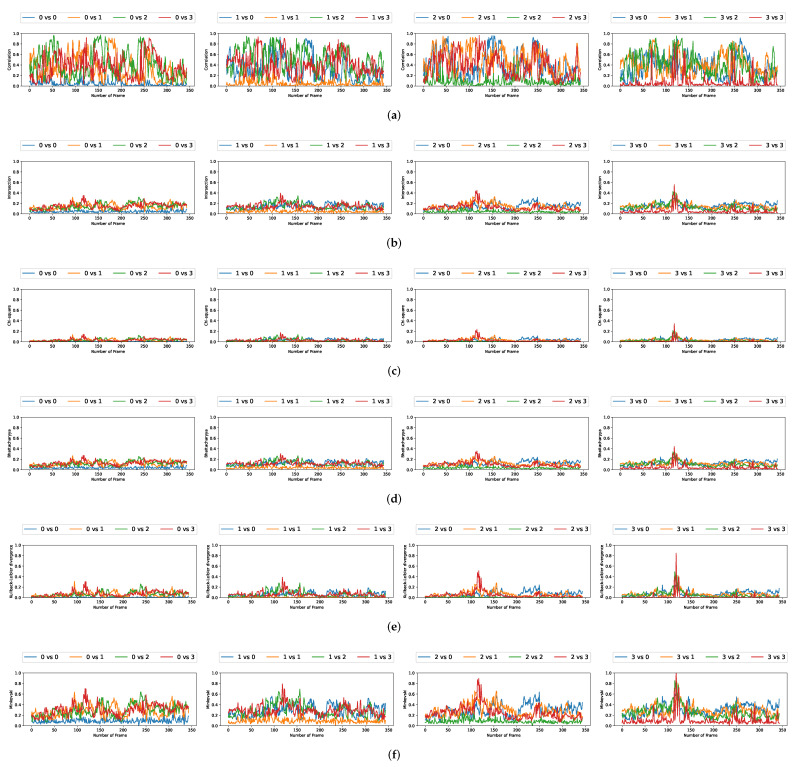
Curves comparing persons from one frame to another in video 1 (persons 0, 1, 2, and 3) using the HOG. (**a**) Correlation. (**b**) Intersection. (**c**) Chi-square. (**d**) Bhattacharyya. (**e**) KL divergence. (**f**) Minkovski.

**Figure 9:**
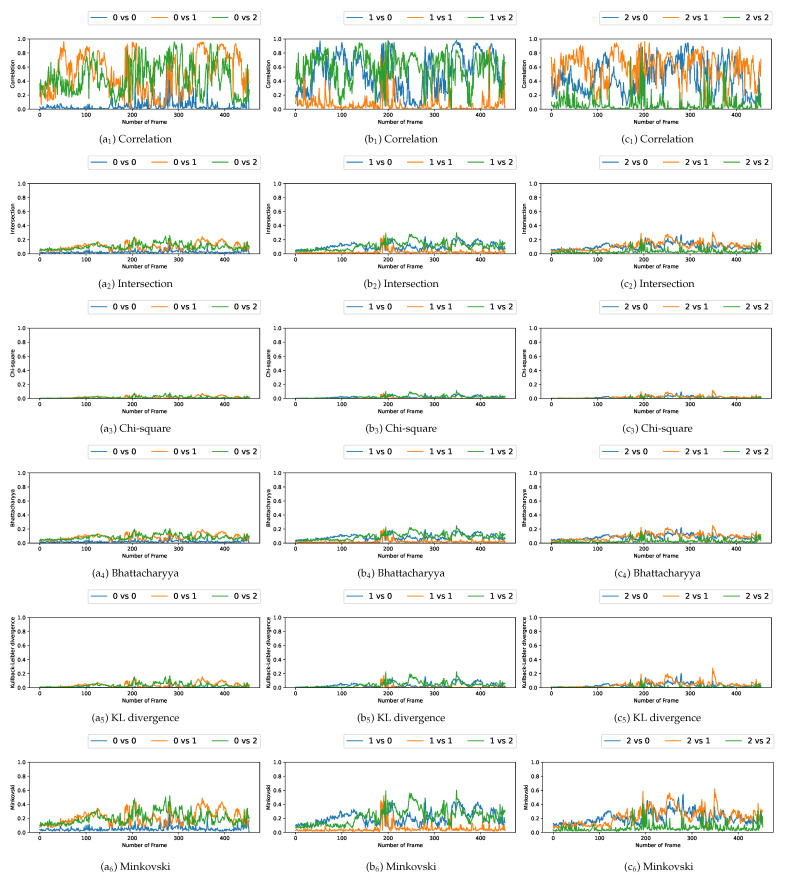
Curves comparing persons from one frame to another in video 2 (persons 0, 1, and 2) using the HOG.

**Figure 10:**
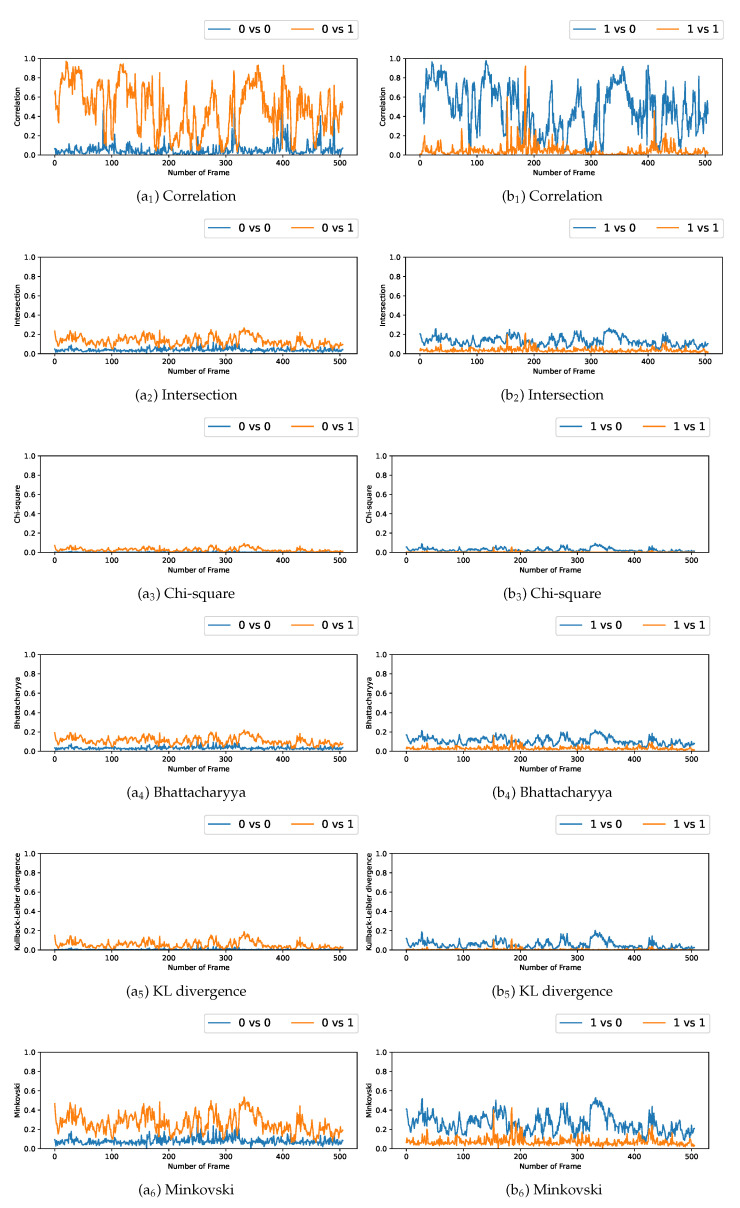
Curves comparing persons from one frame to another in video 3 (persons 0 and 1) using the HOG.

**Figure 11:**
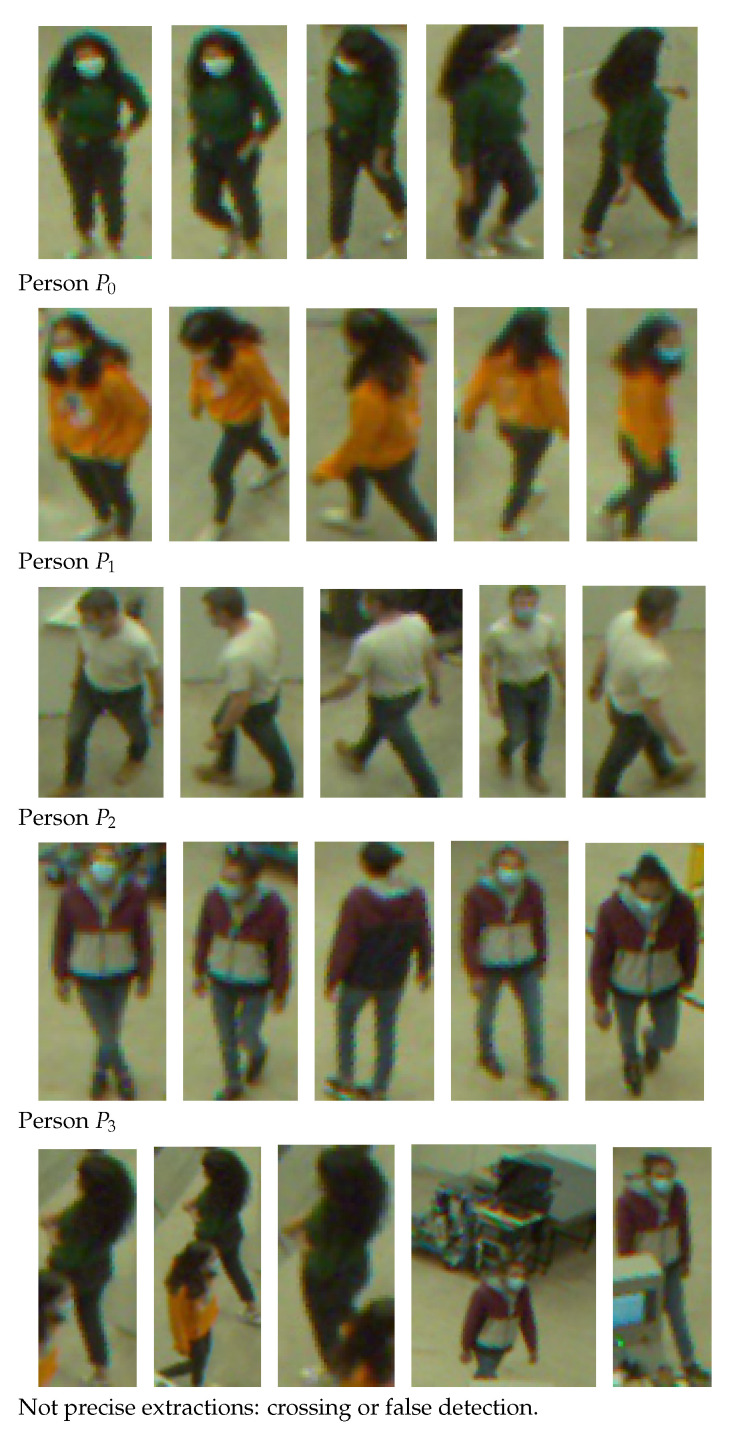
Extractions of the four persons from the first video (namely $P_{i,i\in \{0,1,2,3\}}$) and extractions where there is more than one person (bottom). Note that images are unwrapped for displaying and of different sizes.

We plotted the graphs corresponding to the similarity scores of the HOG and the different color spaces for a comparison with the four persons in the first video. We have numbered the four persons from zero to three. For a given video frame and for a person i∈{0,1,2,3}, we compute the frame by frame distance between its histogram and the four persons of the following frame. By repeating the experiment on all the frames in the video, we obtain a graph composed of four curves that characterizes a person and the color space/distance or HOG/distance couple. This way, we obtain four graphs per couple for the first video. We repeat the process for the two other videos that have two and three people. A part of these graphs is presented in Figure 5, Figure 6, Figure 7, Figure 8, Figure 9 and Figure 10. We can see that for a given graph, which represents the comparison of a person *i* (i∈{0,1,2,3}), one of the curves is lower than the others, it is the one that represents the comparison of this person *i* with itself in the following frame. However, we can sometimes observe some spikes, at frames 160 for the graphs comparing persons 1 and 0 (a${}_{1}$ or b${}_{5}$ as an example). These spikes can be explained by the intersection of the bounding boxes in some frames. We can observe this in Figure 11, where these bounding boxes are displayed; person 0 appears in the bounding box of person 2 (crossing). Different experiments have been carried out after having compared the people in three different videos, but all the curves tied to the different spaces and with the different similarity measures cannot be displayed due to limited space. The following subsection presents an evaluation of the different comparisons. The goal now is to find the best associations of color space(s), see Table 1 or for the HOG and distance(s) *d*, see Table 2. Consequently, the data of the curves must be therefore compared between them.

### 5.4. Evaluating the Comparisons

In this part, the aim is to evaluate which association of the similarity measure with the HOG or color space is the most efficient one to track people. To do so, a metric S is proposed, which is a normalized measure to determine the best among the possible associations.

As mentioned earlier, the underlying objective of this paper is to track people in a video based on the color features. Let us take the example of a video showing two people. Let $P_{1}^{t}$ be the person $P_{1}$ we want to follow in the frame at time *t*, and $P_{2}^{t}$ be the second person $P_{2}$ detected in the same frame. In the next frame, at t+1, let $P_{1}^{t+1}$ and $P_{2}^{t+1}$ correspond to the detection of $P_{1}$ and $P_{2}$, respectively. If the distance between the histograms of $P_{1}^{t}$ and $P_{1}^{t+1}$ is the smallest, then $P_{1}^{t+1}$ is indeed the person $P_{1}$ we want to follow. On the other hand, $P_{2}^{t+1}$ corresponds to the person $P_{1}$ if the distance between the histograms of $P_{1}^{t}$ and $P_{2}^{t+1}$ is the smallest. As a result, the most efficient association of color space and distance is the one that minimizes the distance $P_{1}P_{1}$ between the histograms of the detection of person $P_{1}$ between two frames but maximizes the distance $P_{1}P_{2}$ between the histograms of the detection of persons $P_{1}$ and $P_{2}$ between the two frames.

To determine the best association(s), we computed a quantitative score for each video defined as follows for two persons:

(11) $$S=\frac{inter-distance}{intra-distance}=\frac{\hat{d}\left(H_{P_{1}}H_{P_{1}}\right)+\hat{d}\left(H_{P_{2}}H_{P_{2}}\right)}{2\cdot \hat{d}\left(H_{P_{1}}H_{P_{2}}\right)},$$

where $H_{P_{i}}$ and $H_{P_{j}}$ represent the color histograms of $P_{i}$ and $P_{j}$, respectively, and $\hat{d}$ is the average of a distance *d* (the distances are listed in Table 2) along the video between two consecutive frames. This function can be generalized for *n* sub-images (targets) present in the video as follows:

(12) $$\begin{array}{cc} S & =\sum \frac{inter-distance}{intra-distance}\\ \; & =\frac{2}{n\cdot (n-1)}\cdot \sum_{(i,j),i\neq j}\frac{\hat{d}\left(H_{P_{i}}H_{P_{i}}\right)+\hat{d}\left(H_{P_{j}}H_{P_{j}}\right)}{2\cdot \hat{d}\left(H_{P_{i}}H_{P_{j}}\right)}. \end{array}$$

In this paper, we focused on videos depicting two, three, and four persons so, (i,j) ∈ ${\left\{0,1,2,3\right\}}^{2}$.

Note that the lower the curve comparing the same two people (from different frames) and the farther away from the others. Consequently, lower will be the score given by the S function implying a better association between the color space and the distance.

Table 5 shows the S scores for all the combinations of color spaces with distances in the video with 4 people. Thus, the similarities between the scores are more visible by distance and not by the color space used. The S score values range between 0.11 and 0.45. Theoretically, the values of the function S could range from 0 to infinity, but in practice, they will very rarely exceed 1. One distance stands out for this video: the KL divergence, as it has a really good score in association with CIE L*a*b and CIE L*u*v* color spaces, but the best association is with YCbCr.

Table 6 shows the S score for all the combinations of color spaces with distances in the video of about two people. Once again, the scores are quite similar when the same distance is computed rather than the same color spaces. S ranges between 0.04 and 0.41. Overall, associations with the correlation distance have a low score; most of them at 0.12, but the Kullback–Leibler (KL) divergence has still the lowest scores. The best associations, in this case, are the YCbCr color space with the KL divergence.

Table 7 shows the S score for all the combinations of color spaces with distances in the video with 3 people. Like for the two other videos, the scores are quite similar when the same distance is computed rather than the same color spaces. S ranges between 0.16 and 0.34. Overall, associations with the correlation, chi-square, and the Kullback–Leibleir divergence have a low score, Ultimately, the association between CIE L*u*v and correlation is the best with a score of 0.09.

One can notice that, compared to the results obtained by using the color spaces, the S scores resulting from the use of the HOG are not that enviable, as a whole. Nevertheless, some scores are even among the best; we must keep in mind that this function S has its limits as it is defined using averages. Consequently, when the overall results of the distance functions are close to 0—this is particularly the case concerning the HOG—the S score is also close to 0. For regular results, it is a good estimator, but as soon as we get more fluctuating results, the S function loses some sense, and this is unfortunately what we can observe in Figure 8, Figure 9 and Figure 10 in the correlation curves, for example.

## 6. Conclusions

Throughout this paper, the effectiveness of different color spaces and distance pairings for images from a fisheye camera has been evaluated. This study highlights one similarity function, particularly the Kullback–Leibler (KL) divergence. Indeed, this distance obtains the best S score. This can be explained by the fact that its value can be greater than 1; consequently, two very different images can be more easily differentiated. KL divergence works the best with YCbCr space, this combination gives good results in almost all cases, even though it also gives interesting results with CIE L*a*b* and CIE L*u*v* color spaces. Note that some other associations give favorable results such as correlation and chi-square with YCbCr, CIE L*a*b* and CIE L*u*v* or KL divergence with RGB. The HOG can nevertheless obtain very good results, its use is to be seriously considered according to what one seeks; it is necessary nevertheless to keep in mind the limits of the S scores. This study can be useful for works related to tracking. It gives the preferred combination when using a fisheye camera, which is a sensor providing strong deformations in the image. This study was limited to six color spaces and six distances, but it would be interesting to push it further by evaluating more distances and more elaborate color spaces [17]. In addition, we noticed that the RAPiD method was not always 100% accurate since the bounding boxes were sometimes crossed and the background of the detected images could disturb the distance evaluation. It could be interesting to look for and use a more accurate algorithm that would limit the region of interest encompassed by the detected person (its shape) and therefore eliminate the background, optimally. Converting to certain spaces is sometimes time consuming, and so is evaluating distances between two histograms. It would be interesting to see if the reduction(s) of the size of the images or of the histograms cause(s) a loss in precision by bringing a gain in time.

## Figures and Tables

**Table 1:** Typical color spaces with their acronyms and range in OpenCV software for histogram computation.

Space’s Name	Components	Range
	R (Red)	[0, 255]
RGB	G (Green)	[0, 255]
	B (Blue)	[0, 255]
	H (Hue)	[0, 180]
HSV	S (Saturation)	[0, 255]
	V (Value)	[0, 255]
	H (Hue)	[0, 180]
HLS	L (Lightness)	[0, 255]
	S (Saturation)	[0, 255]
	L* (Lightness)	[0, 255]
CIE L*a*b*	a* (Red/green coordinate)	[0, 255]
	b* (Yellow/blue coordinate)	[0, 255]
	L* (Lightness)	[0, 255]
CIE L*u*v*	u* (Red/green coordinate)	[0, 255]
	v* (Yellow/blue coordinate)	[0, 255]
	Y (Luminance)	[0, 255]
YCbCr	Cr (Red-difference)	[0, 255]
	Cb (Blue-difference)	[0, 255]

**Table 2:** Distance measures to compare 2 different histograms $H_{1}$ and $H_{2}$ of the same length. Usually, $H_{1}$ and $H_{2}$ are both normalized.

Distance Metrics	Equation
Correlation	$d\left(H_{1},H_{2}\right)=\frac{1}{2}\cdot \left(1-\frac{\sum_{k=0}^{N-1}\left(H_{1}\left(k\right)-\bar{H_{1}}\right)\cdot \left(H_{2}\left(k\right)-\bar{H_{2}}\right)}{\sqrt{\sum_{k=0}^{N-1}{\left(H_{1}\left(k\right)-\bar{H_{1}}\right)}^{2}\cdot \sum_{k=0}^{N-1}{\left(H_{2}\left(k\right)-\bar{H_{2}}\right)}^{2}}}\right)$
Chi-square $\left(\chi^{2}\right)$	$d\left(H_{1},H_{2}\right)=\sum_{k=0}^{N-1}\frac{{\left(H_{1}\left(k\right)-H_{2}\left(k\right)\right)}^{2}}{2\cdot (H_{1}\left(k\right)+H_{2}\left(k\right))}$
Intersection	$d\left(H_{1},H_{2}\right)=1-\sum_{k=0}^{N-1}min\left(H_{1}\left(k\right),H_{2}\left(k\right)\right)$
Bhattacharyya	$d\left(H_{1},H_{2}\right)=\sqrt{1-\frac{1}{\sqrt{\bar{H_{1}}\cdot \bar{H_{2}}\cdot N^{2}}}\sum_{k=0}^{N-1}\sqrt{H_{1}\left(k\right)\cdot H_{2}\left(k\right)}}$
KL divergence	$d\left(H_{1},H_{2}\right)=\sum_{k=0}^{N-1}H_{1}\left(k\right)\cdot log\left(\frac{H_{1}\left(k\right)}{H_{2}\left(k\right)}\right)$
Manhattan distance	$d\left(H_{1},H_{2}\right)=\sum_{k=0}^{N-1}\left|H_{1}\left(k\right)-H_{2}\left(k\right)\right|$

**Table 3:** Example of comparison of distance scores between several persons in two different frames as a function of the color space. The reference image on the third line is extracted in the image presented in Figure 2b whereas other persons are tied to the detected persons in Figure 2a. Note that images are unwrapped for displaying.

		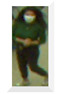 Person P1 at Time t+x						
Image at Time *t*	Distance	RGB	HSV	HLS	L*a*b*	L*u*v*	YCrCb	HOG
**Person P0**	Correlation	0.27	0.14	0.13	0.09	0.09	0.1	0.48
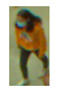	Chi-square $\left(\chi^{2}\right)$	0.2	0.17	0.15	0.18	0.18	0.2	0.03
	Intersection	0.38	0.32	0.31	0.33	0.33	0.35	0.1
	Bhattacharyya	0.34	0.32	0.3	0.35	0.35	0.37	0.08
	Kullback–Leibler	0.58	0.51	0.43	0.44	0.47	0.5	0.05
	Minkovski	0.76	0.65	0. 63	0. 66	0.72	0.7	0.19
**Person P1**	Correlation	0.18	0.08	0.07	0.05	0.05	0.05	0.45
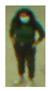	Chi-square $\left(\chi^{2}\right)$	0.14	0.09	0.09	0.1	0.1	0.1	0.06
	Intersection	0.28	0.22	0.22	0.22	0.22	0.22	0.18
	Bhattacharyya	0.31	0.25	0.24	0.26	0.26	0.27	0.18
	Kullback–Leibler	0.38	0.29	0.3	0.25	0.29	0.26	0.15
	Minkovski	0.57	0.45	0.45	0.43	0.47	0.45	0.36
**Person P2**	Correlation	0.27	0.1	0.11	0.05	0.05	0.06	0.7
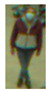	Chi-square $\left(\chi^{2}\right)$	0.19	0.12	0.13	0.11	0.11	0.12	0.04
	Intersection	0.35	0.26	0.27	0.24	0.24	0.26	0.14
	Bhattacharyya	0.34	0.29	0.29	0.26	0.27	0.27	0.15
	Kullback–Leibler	0.65	0.66	0.8	0.33	0.43	0.38	0.09
	Minkovski	0.71	0. 53	0.54	0.47	0.54	0.53	0.27
**Person P3**	Correlation	0.24	0.08	0.08	0.04	0.04	0.05	0.6
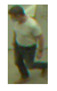	Chi-square $\left(\chi^{2}\right)$	0.18	0.11	0.1	0.09	0.09	0.1	0.08
	Intersection	0.36	0.26	0.24	0.23	0.23	0.25	0.22
	Bhattacharyya	0.33	0.26	0.26	0.23	0.23	0.24	0.25
	Kullback–Leibler	0.67	0.48	0.64	0.29	0.41	0.33	1.53
	Minkovski	0.73	0.53	0.49	0.46	0.5	0.49	0.45

**Table 4:** Main characteristics of the applied database.

	Number of Images	Frame Rate	Where	Number of People to Track
Video 1	535	40	Hall	4
Video 2	535	40	Hall	2
Video 3	535	40	Classroom	3

**Table 5:** S scores calculated for the 1st video with 4 people.

Color Spaces or Descriptor	Correlation	Chi-Square $\left(\chi^{2}\right)$	Intersection	Bhattacharyya	Kullback–Leibler	Manhattan
RGB	**0.14**	0.16	0.36	0.39	0.16	0.38
HSV	0.18	0.22	0.42	0.45	0.22	0.44
HLS	0.17	0.22	0.41	0.45	0.22	0.43
CIE L*a*b*	0.16	**0.14**	0.35	0.35	**0.12**	0.36
CIE L*u*v*	0.15	**0.14**	0.34	0.35	**0.12**	0.35
YCbCr	**0.14**	**0.13**	0.33	0.34	**0.11**	0.35
HOG	0.17	0.16	0.34	0.35	0.16	0.34

**Table 6:** S scores calculated for the 2nd video with 2 people.

Color Spaces or Descriptor	Correlation	Chi-Square $\left(\chi^{2}\right)$	Intersection	Bhattacharyya	Kullback–Leibler	Manhattan
RGB	**0.12**	0.14	0.34	0.35	**0.11**	0.34
HSV	**0.12**	0.18	0.38	0.40	0.14	0.38
HLS	**0.12**	0.19	0.38	0.41	0.15	0.38
CIE L*a*b*	0.14	0.13	0.33	0.32	**0.06**	0.33
CIE L*u*v*	**0.12**	**0.12**	0.31	0.31	**0.05**	0.31
YCbCr	**0.12**	**0.11**	0.30	0.30	**0.04**	0.30
HOG	0.11	0.21	0.59	0.60	0.20	0.59

**Table 7:** S scores calculated for the 3rd video with 3 people.

Color Spaces or Descriptor	Correlation	Chi-Square $\left(\chi^{2}\right)$	Intersection	Bhattacharyya	Kullback–Leibler	Manhattan
RGB	0.20	0.21	0.31	0.33	**0.16**	0.32
HSV	0.20	0.21	0.33	0.34	0.19	0.33
HLS	0.20	0.21	0.33	0.35	0.20	0.33
CIE L*a*b*	0.19	0.20	0.30	0.32	0.18	0.30
CIE L*u*v*	0.19	0.19	0.29	0.30	**0.17**	0.29
YCbCr	0.19	0.20	0.29	0.31	**0.17**	0.29
HOG	**0.13**	**0.12**	0.27	0.28	**0.12**	0.27

## Data Availability

The data presented in this study can be found here: https://partage.imt.fr/index.php/s/nytmFqiq8jaztkX (accessed on 28 March 2022).

## Appendix A

To realize our experiments, we started with images recorded in the RGB color space. In order to test the various spaces of colors, it is necessary to convert these images into the other spaces by the mean of different combinations. Table A1 summarizes the equations which are provided in the OpenCV documentation; they allow passing from RGB space to all the other spaces studied in this paper. Of course, there exist equations computing the inverse and recovering RGB space from the other spaces of colors or passing from any space of color to another, see [17]. We can note in the equations of Table A1 that values of each channel R, G, B are considered to be between 0 and 1. Nevertheless, the inputs are 8-bit images, so the range of each channel is between 0 and 255; it is necessary to simply normalize the different channels as follows:

$$R=\frac{R}{255\prime }\;G=\frac{G}{255\prime }\;B=\frac{B}{255}.$$

Otherwise, without this normalization, there may be information loss for conversion from linear to non-linear spaces such as for RGB to L*u*v* or L*a*b transformations.

**Table A1:** Usual color spaces computed from RGB space.

Color Spaces	Equation
HSV	V ←max(R,G,B)
	S ← $\left\{\begin{array}{cc} \frac{V-min(R,G,B)}{V} & \mathrm{if}\;V\neq 0\\ 0 & \mathrm{otherwise} \end{array}\right.$
	H ← $\left\{\begin{array}{cc} \frac{60(G-B)}{V-min(R,G,B)} & \mathrm{if}\;V=R\\ \frac{120+60(B-R)}{V-min(R,G,B)} & \mathrm{if}\;V=G\\ \frac{240+60(R-G)}{V-min(R,G,B)} & \mathrm{if}\;V=B\\ 0 & \mathrm{if}\;V=R=G=B \end{array}\right.$
HLS	$V_{max}\leftarrow max\left(R,G,B\right)$
	$V_{min}\leftarrow min\left(R,G,B\right)$
	L $\leftarrow \frac{V_{max}+V_{min}}{2}$
	S ← $\left\{\begin{array}{cc} \frac{V_{max}-V_{min}}{V_{max}+V_{min}} & \mathrm{if}\;L<0.5\\ \frac{V_{max}-V_{min}}{2-V_{max}+V_{min}} & \mathrm{if}\;L\geq 0.5 \end{array}\right.$
	H ← $\left\{\begin{array}{cc} \frac{60(G-B)}{V_{max}-V_{min}} & \mathrm{if}\;V_{max}=R\\ \frac{120+60(B-R)}{V_{max}-V_{min}} & \mathrm{if}\;V_{max}=G\\ \frac{240+60(R-G)}{V_{max}-V_{min}} & \mathrm{if}\;V_{max}=B\\ 0 & \mathrm{if}\;V_{max}=R=G=B \end{array}\right.$
YCbCr	Y ←0.299R+0.587G+0.114B
	Cr ←(R−Y)0.713+δ
	Cb ←(B−Y)0.564+δ
	whereδ=128(Inthecaseof8−bitsimages)
CIE L*a*b	$\left(\begin{array}{c} X\\ Y\\ Z \end{array}\right)$$\leftarrow \left(\begin{array}{ccc} 0.412453 & 0.357580 & 0.180423\\ 0.212671 & 0.715160 & 0.072169\\ 0.019334 & 0.119193 & 0.950227 \end{array}\right)$ . $\left(\begin{array}{c} R\\ G\\ B \end{array}\right)$
	X $\leftarrow \frac{X}{X_{n}}\;\mathrm{where}\;X_{n}=0.950456$
	Z $\leftarrow \frac{Z}{Z_{n}}\;\mathrm{where}\;Z_{n}=1.088754$
	L ← $\left\{\begin{array}{cc} 116Y^{1/3}-16 & \mathrm{if}\;Y>0.008856\\ 903.3Y & \mathrm{if}\;Y\leq 0.008856 \end{array}\right.$
	a ←500(f(X)−f(Y))+δ
	b ←200(f(Y)−f(Z))+δ
	wheref= $\left\{\begin{array}{cc} t^{1/3} & \mathrm{if}\;t>0.008856\\ 7.787t+\frac{16}{116} & \mathrm{if}\;t\leq 0.008856 \end{array}\right.$
	andδ=128(Inthecaseof8−bitsimages)
CIE L*u*v	$\left(\begin{array}{c} X\\ Y\\ Z \end{array}\right)$$\leftarrow \left(\begin{array}{ccc} 0.412453 & 0.357580 & 0.180423\\ 0.212671 & 0.715160 & 0.072169\\ 0.019334 & 0.119193 & 0.950227 \end{array}\right)$ . $\left(\begin{array}{c} R\\ G\\ B \end{array}\right)$
	L ← $\left\{\begin{array}{cc} 116Y^{1/3}-16 & \mathrm{if}\;Y>0.008856\\ 903.3Y & \mathrm{if}\;Y\leq 0.008856 \end{array}\right.$
	$u^{\prime }\leftarrow \frac{4X}{X+15Y+3Z}$
	$v^{\prime }\leftarrow \frac{9Y}{X+15Y+3Z}$
	u $\leftarrow 13L\left(u^{\prime }-u_{n}\right)\;\mathrm{where}\;u_{n}=0.19793943$
	v $\leftarrow 13L\left(v^{\prime }-v_{n}\right)\;\mathrm{where}\;v_{n}=0.46831096$
